# Using 2*k* + 2 bubble searches to find single nucleotide polymorphisms in *k*-mer graphs

**DOI:** 10.1093/bioinformatics/btu706

**Published:** 2014-10-24

**Authors:** Reda Younsi, Dan MacLean

**Affiliations:** The Sainsbury Laboratory, Norwich Research Park, Norwich NR4 7UH, UK

## Abstract

**Motivation:** Single nucleotide polymorphism (SNP) discovery is an important preliminary for understanding genetic variation. With current sequencing methods, we can sample genomes comprehensively. SNPs are found by aligning sequence reads against longer assembled references. De Bruijn graphs are efficient data structures that can deal with the vast amount of data from modern technologies. Recent work has shown that the topology of these graphs captures enough information to allow the detection and characterization of genetic variants, offering an alternative to alignment-based methods. Such methods rely on depth-first walks of the graph to identify closing bifurcations. These methods are conservative or generate many false-positive results, particularly when traversing highly inter-connected (complex) regions of the graph or in regions of very high coverage.

**Results:** We devised an algorithm that calls SNPs in converted De Bruijn graphs by enumerating 2*k* + 2 cycles. We evaluated the accuracy of predicted SNPs by comparison with SNP lists from alignment-based methods. We tested accuracy of the SNP calling using sequence data from 16 ecotypes of *Arabidopsis thaliana* and found that accuracy was high. We found that SNP calling was even across the genome and genomic feature types. Using sequence-based attributes of the graph to train a decision tree allowed us to increase accuracy of SNP calls further. Together these results indicate that our algorithm is capable of finding SNPs accurately in complex sub-graphs and potentially comprehensively from whole genome graphs.

**Availability and implementation:** The source code for a C++ implementation of our algorithm is available under the GNU Public Licence v3 at: https://github.com/danmaclean/2kplus2. The datasets used in this study are available at the European Nucleotide Archive, reference ERP00565, http://www.ebi.ac.uk/ena/data/view/ERP000565

**Contact:** dan.maclean@tsl.ac.uk

**Supplementary information:** Supplementary data are available at *Bioinformatics* online.

## 1 Introduction

**Fig. 1.**
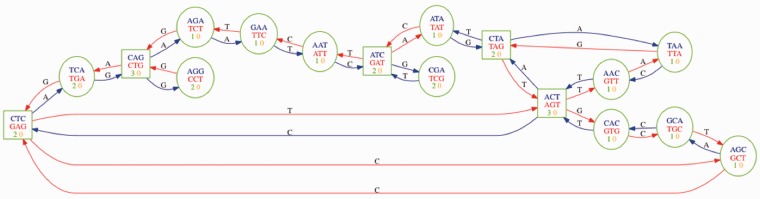
De Bruijn graph fragment with edge and vertex attributes as represented in the Cortex software and used in this study. Vertices represent *k*-mers from sequence fragments and their reverse complements, edges represent directional overlap between sequence and edge annotations represent the changed base between edges. Coloured numbers in vertices indicate coverage (times the *k*-mer is observed) in the respective ’orange’ or ’green’ coloured sample

Single nucleotide polymorphisms (SNPs) between genomes of individuals are valuable markers for tracing the genetic basis of inheritable traits or diseases. Rapid detection and creation of large libraries of SNPs is vital for timely investigation and identification of genes associated with important phenotypes. Contemporary sequencing technology can sample genomes comprehensively in only hours, with these data SNP detection is typically achieved by aligning reads to a reference genome and identifying SNPs as a difference between consensus and the reference (Leggett and MacLean, 2014). Factors such as the need for a reference sequence and the assumption of a monomorphic sample mean that the consensus approach is limited in organisms for which we lack a reference genome, in outbred diploid samples, bulked population data or analysis of metagenomes. To deal with the very large amount of sequence data that current sequencing technologies produce, De Bruijn graphs have been used to represent *k*-mer (nucleotide subsequences of arbitrary length *k*) overlap patterns in sequence reads. These *k*-mer graphs have been implemented into efficient data structures for large collections of *k*-mers and have proven to be of great utility as the underlying data model over which numerous *de novo* genome assembly algorithms have been implemented (Pevzner *et* *al*., 2001, Zerbino and Birney, 2008, Simpson *et* *al*., 2009). Methods based on the De Bruijn graph have been implemented recently that can efficiently identify sample differences in sequence read sets (Iqbal *et* *al*., 2012, Leggett *et* *al*., 2013). Iqbal et al. (2012) produced the first model and *de novo* assembly algorithms for variant discovery and genotyping directly from sequence data without using a reference genome. They incorporated a colour attribute for the edges in the graph that represents the sample from which the sequence is derived. In these multi-sample graphs polymorphisms appear as bubbles, a closing bifurcation of length 2*k* + 2 with separate colours on each branch of the bifurcation 1. Discovering bubbles is a promising tactic for identifying SNPs in the graph, the bubble caller in Iqbal *et al.* and the more computationally intensive depth-first search method in Leggett *et al.* (2013) proceeds by marking the vertices in the graph that have at least two edges departing from them as starting points and following vertices sequentially until a vertex with at least two edges entering it is reached (Fig. 1). An alternative micro-assembly approach (Peterlongo *et* *al*., 2010) begins by producing a tree of *k*-mers for an input read set picking a seed *k*-mer and assuming that it lies on one path through a SNP and then looks for an opposite *k*-mer, one substitution different, which would lie on another path through the bubble. If this can be found in the *k*-mer tree, then a recursive algorithm builds paths left and right of each *k*-mer until they join or no *k*-mer can be found. Further to graph structure, the attributes of the sample and sampled sequence reads can be used.

## 2 Approach

De Bruijn graphs of sequence reads are typically very large with the vast majority of vertices having only a single in and out edge, as such no complex bubbles can exist in these areas and much of the graph need not be searched. Thus, we first build a sub-graph from a branching vertex that has at least two out edges and search sub-graphs for cycles. We retain only cycles of length 2*k* + 2 with two equidistant branching vertices in which the edge paths on each branch are a different colour. Internal nodes may be branching. These constraints allow us to simplify the search for cycles by only checking the locality of the starting branching vertex. Once the algorithm terminates, a simple walk of the vertices in the bubble, collecting the labels, gives the sequence. The two coloured *k* + 1 walks result in separate nucleotide sequences of length *k* + 1 in which the first nucleotide differs between the samples. Our method’s very precise description of a bubble means that it has the potential to be extremely specific and generate highly accurate lists of SNPs from graph structure alone.

## 3 Methods

### 3.1 Datasets

We used thirteen sets of Illumina sequence reads from different ecotypes of the model plant *A.thaliana* with the *A.thaliana* ecotype Col-0 genome as the common reference (AGI, 2000). For each of these, high-quality SNP lists are available at the European Nucleotide Archive under reference ERP000565 (Gan *et al*., 2011). Reads are of 36 and 51 bp long with 200- and 400-bp inserts, respectively, with between 27- and 60-fold coverage. *Arabidopsis thaliana* has a small (126 Mbp) tractable genome with only a relatively small repeat content and well-catalogued genetic diversity, making it an ideal test organism.

### 3.2 2*k* + 2 Bubble detection algorithm

We used Cortex (Iqbal *et* *al*., 2012) to build a graph directly from sequence data, removing paths of vertices with coverage 2 or below and tips less than 100 nucleotides in length. The resulting graph is exported and is then transformed to an undirected graph, retaining the edge attributes. Then 2*k* + 2 (

**Algorithm 1. A bubble detection algorithm (2*k* + 2)**

1. *G* is an undirected graph; G=(V,E), edges have attributes *sequence* and colour∈(orange,green,mixed)
2. *T* is the maximum number of sub-graphs to be created, T∈N
3. *S* is an initially empty set of found cycles, S=⊘
4. *C* is an initially empty set of computed contigs, C=⊘
5. *k* is the length of the string in the *sequence* edge attribute, k∈N
6. *b* is a list of all branching vertices in *G*
7. **while** t≠T **do**
8.  Build sub-graph g⊂G
9.  Select a vertex *v* from *b*
10.  Traverse 2*k* + 2 edges in *g*, store vertices passed as *s*
11.  **if** *v* is the current vertex **and** *s* has 2 equidistant branching vertices with distinct variant colours **then**
12.    S←s
13.  **end if**
14. **end while**
15. **for** *s* in *S* **do**
16.  **for** *colour* in (*orange*, *green*, *mixed*) **do**
17.    *c* is empty string
18.    Traverse edges of *s* that have attribute *colour*
19.    Append last character from edge attribute string *sequence* to *c*
20.  **end for**
21.  C←c
22. **end for**
23. Output: Set of contigs *C*

Algorithm 1) is applied to create short contigs bearing SNPs. The search is based around identifying sub-graphs to search. The size of the sub-graphs is user defined and chosen depending on the complexity of the graph. A small sub-graph in a simple graph structure can return no SNPs and a sub-graph in a very complex structure has a prohibitive running time, there is no algorithmic bound. The algorithm as a whole is fast and can be easily modified for parallel processing. For the datasets used in this study searches ran from 4 to 48 h depending on the structure of the graph. In addition, the algorithm has a free parameter to choose the number of predicted SNPs to call and a time limit parameter to run. The algorithm allows flexible graph search strategies and by cutting the graphs into sub-graphs using a neighbourhood technique, we can speed up the search process and make the search extremely parallel, and therefore, potentially extremely scalable.

### 3.3 Using canonical SNP lists to assess accuracy of the algorithm

To assess the accuracy of the SNPs predicted by 2*k* + 2, we compared our predictions against published lists of SNPs called in the Xiangchao et al. (2011) study. The contigs generated by the 2*k* + 2 algorithm were used as query sequence in a BLASTn search with default settings (Altschul *et* *al*., 1990) against the *A.thaliana* Col-0 reference sequence and used the top hit in the BLASTn to find the position of the predicted SNP in the genome. By comparing the position of the published SNPs with those from our algorithm, we could then calculate the number of SNPs accurately predicted by our algorithm as the number found in the published lists, accuracy is calculated as proportion of predicted SNPs that were included in the known SNP list. We used three measures of accuracy; sensitivity (true positive/true positive + false negative), specificity (true negative/true negative + false positive) and accuracy (proportion of predicted SNPs that were included in the known SNP list). Specificity in this analysis is always very close to 100% because of the very large number of non-SNP genome sites. As our algorithm searches only a subset of the graph defined in parameter *T* (Algorithm 1), we do not search the whole graph. In experiments here, we search a maximum of 200 000 sub-graphs and call accuracy on the number of SNPs retrieved in that set, not the whole graph.

### 3.4 Using bubble attributes to filter and improve accuracy

To use a classification algorithm such as decision trees (Quinlan, 1986), 2*k* + 2 bubble edge attributes were used to create a vector of values describing each bubble. We used coverage, summed coverage over each branch of the bubble and the mean number of branching vertices of the sub-graph where the bubble resides (See Supplementary Table S1 for summaries of the datasets used for classification). Each bubble in a graph was classified as a Real SNP or a false positive according to its presence in the SNP list and were divided into a training set and testing set. Two-third of the dataset is used for training the classifier and one third for testing. To train, we used a decision tree found in the freely available WEKA package (Hall, 2009) and kept the default parameters of the classifier.

## 4 Results and discussion

### 4.1 SNP prediction based on structure alone is highly accurate.

**Fig. 2.**
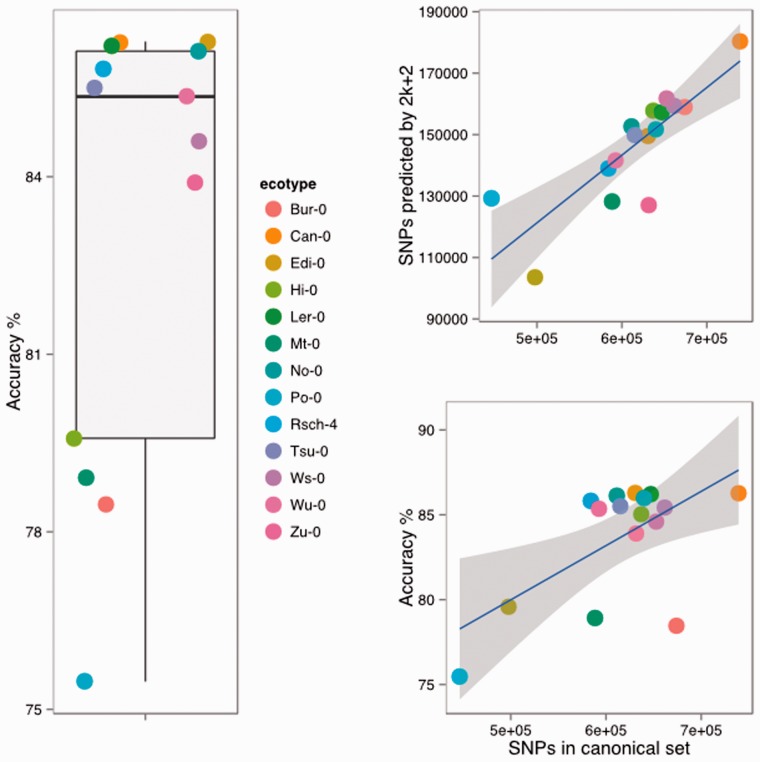
Accuracy of SNP prediction using the 2*k* + 2 algorithm on 13 *A.thaliana* ecotypes. Panel A (left side) shows distribution of accuracy over all 16 ecotypes. Panel B (top right) gives the number of SNPs predicted found by 2*k* + 2 as a function of the SNPs expected from the canonical list. Panel C (bottom right) gives the number of SNPs accurately predicted as a function of the number of SNPs in the canonical set

We ran the 2*k* + 2 algorithm on graphs built with *k* = 21 and predicted SNPs in the thirteen *A.thaliana* ecotypes. On average, we predicted 144541.6146741.9 ± 19560.318183.2 SNPs. The total number of SNPs we predicted increases fairly linearly with the genetic distance of the test ecotype from the reference Col-0 (using the number of canonical SNPs between Col-0 and each ecotype as a proxy for distance, Fig. 2B) and we achieved mean accuracy of 83.2783.7 ± 3.83.5% of correctly predicted SNPs (Fig. 2B), indicating that bubble retrieval with our bubble search algorithm alone produces highly accurate SNP predictions and that the algorithm can detect more SNPs when there are more to be detected. The genetic distance of the ecotype from the Col-0 reference does not have a marked effect on accuracy in the majority of cases we examined. For 912 of the ecotypes, we observed accuracy clustered around the 85% mark (Fig. 2C), indicating a natural limit on the accuracy of the prediction when using bubbles. Four of the ecotypes had lower than 80% accuracy. The accuracy is higher than that seen in the unsorted output of the Cortex (Iqbal *et* *al*., 2012) variant caller, which we reported at around 40% but equivalent to that seen when the output from that program is sorted according to the Bubbleparse metric described in Leggett *et* *al*. (2013). Independent runs of a later and improved Cortex on a selection of the datasets used here show higher accuracy (93 ± 0.15%, see Supplementary Data), but recalling a smaller number of the known SNPs than seen in Leggett *et* *al*. (2013) perhaps indicating a tradeoff between recall and accuracy now available in Cortex.

### 4.2 Accuracy of SNP calling is consistent across the genome

**Fig. 3.**
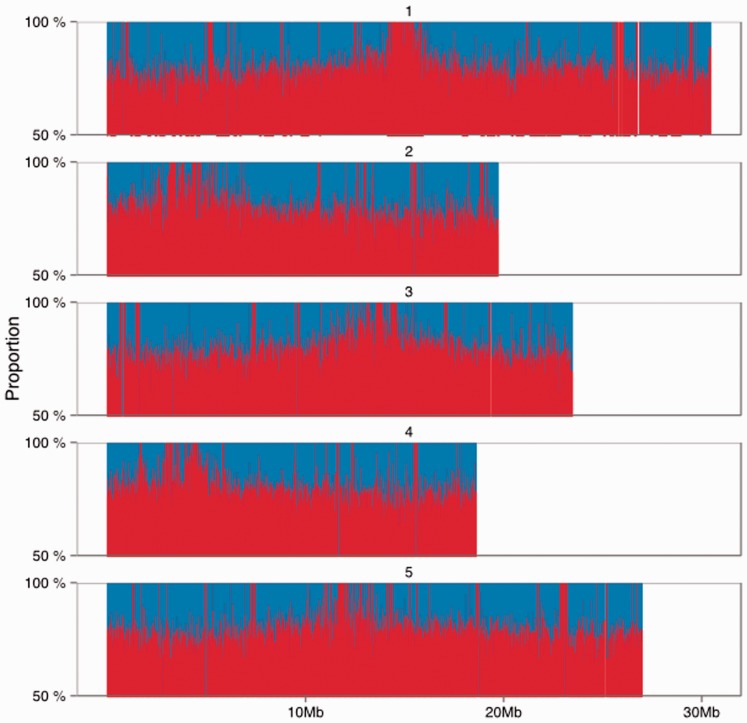
The proportion of consensus-called SNPs predicted by the 2*k* + 2 algorithm in 20 kb windows of the five *Arabidopsis thaliana* nuclear chromosomes SNPs were called on graphs composed of reads from ecotype Tsu-1 relative to the Col-0 common reference. Red peaks indicate the proportion of all SNPs (blue area) found by 2*k* + 2 in each window. Windows reaching 100% likely contain a small number of canonical SNPs, particularly in centromere associated regions

To determine whether our strategy suffered from bias towards particular genomic regions, we compared the position of SNPs predicted by the 2*k* + 2 structure with those from the canonical lists for Col-0 and Tsu-1. Analysis of the proportion of published SNPs found by 2*k* + 2 in windows of 20 kbp across the genome showed consistently high recovery of SNPs. Average recall in the windows was 73% (Fig. 3). The number of published SNPs detected fell substantially in regions corresponding to the centromeres, though the proportion detected increased due to lower numbers of SNPs in these windows.

To determine whether SNPs in any particular genomic feature types were preferentially missed by our algorithm, we examined enrichment of feature types in the 2.5% least sensitive 20 kbp windows. The distribution of accuracy–sensitivity estimates in the windows was observed to be approximately normal, and we applied a Bonferroni-corrected hypergeometric test to each feature type, comparing the proportion in the TAIR 9 Gene Ontology annotation of the genome with the proportion in the bottom 2.5% of accuracy–sensitivity windows. We saw that non-coding regions such as pseudogenes and transposable elements were enriched in the sample (*P* < 0.001; Table 1), it is likely because centromeric regions are enriched in these features (AGI, 2000). The lack of bias towards successful SNP calls in any particular genomic feature or region indicates that the random search strategy we are using to create sub-graphs does not result in bias towards any specific parts of the genome, and we conclude that the 2*k* + 2 algorithm has the potential to be part of a general SNP finding pipeline.

**Table 1.** Genomic feature enrichment in the *A.thaliana* genomic 20 kb windows that had the lowest 2.5% called SNP sensitivity rates in Col-0/Tsu-1 data

Feature	Genome Proportion	Sample Proportion	P
Exon	0.74	0.72	1
5’ UTR	0.12	0.08	1
3’ UTR	0.11	0.11	1
miRNA	0.00	0.00	1
tRNA	0.00	0.00	1
ncRNA	0.00	0.01	<0.001
Pseudogene	0.00	0.01	<0.001
Pseudogenic transcript	0.00	0.01	<0.001
Pseudogenic exon	0.00	0.01	<0.001
Transposable element gene	0.01	0.05	<0.001
snoRNA	0.00	0.00	1
snRNA	0.00	0.00	1
rRNA	0.00	0.00	1

For each feature type, the proportion of all features in the genome and in the sample and a Bonferroni corrected *P*-value from the hypergeometric test is presented.

### 4.3 The 2*k* + 2 algorithm finds SNPs in complex portions of the graph

**Fig. 4.**
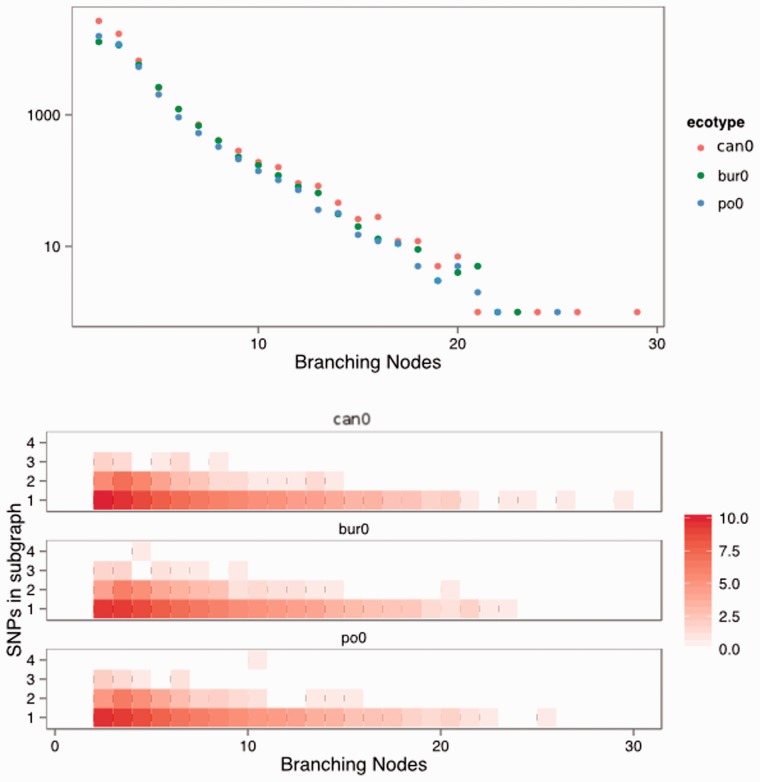
Distribution of number of sub-graphs with given number of branching nodes (A, top panel) and the number of SNPs predicted in all sub-graphs with given number of branching nodes (B, bottom panel). Data from three ecotypes Can-0, Bur-0 and Po-0 are presented. Colour scale represents natural log of counts, see Supplementary Data for real values

To establish the performance of the algorithm in complex regions of the graph, we compared the total number of branching vertices in a 100 vertex sub-graph with the number of SNPs that were predicted when each sub-graph was searched exhaustively. We looked at 54 616, 35 043 and 36 483 randomly sampled sub-graphs with more than two branching vertices for ecotypes Can-0, Bur-0 and Po-0, respectively. The majority of sub-graphs have just a few branching vertices though there are a few very heavily branched sub-graphs with more than 20 out of 100 vertices with branches (Fig. 4A). The number of SNPs predicted in each sub-graph is most usually 1 (49, 36 and 43 of sub-graphs contain only 1 SNP for Can-0, Bur-0 and Po-0, see Supplementary Data) regardless of the number of branching vertices. We would expect the total number of SNPs predicted to increase as graph complexity increases, since SNPs nearby in the genome (in particular within *k* of each other) would increase graph complexity. However, this is not observed, for sub-graphs with more than 15 branching vertices, we predict only one SNP in any ecotype (Fig. 4). Intuitively, we would expect the total number of SNPs predicted to increase as complexity increases as SNPs nearby to each in the genome other would increase graph complexity but this is not observed and for sub-graphs with more than 15 branching vertices, we predict only one SNP in any ecotype 4. Above 90% of predicted SNPs are in sub-graphs with only 2–5 branching vertices (Fig. 4B and Supplementary Data). The result indicates that the algorithm is most successful at retrieving SNPs in sub-graphs with only two branching vertices, though can handle moderately complex graphs and successfully identify SNPs within those sub-graphs. We have not been able to confirm whether SNP density does truly increase with graph complexity, and therefore, cannot rule out whether SNP prediction drop off in more complex regions is a true property of the graph or a failure of the 2*k* + 2 algorithm to detect in these regions.

### 4.4 Accuracy is improved by filtering candidate bubbles using a decision tree

**Fig. 5.**
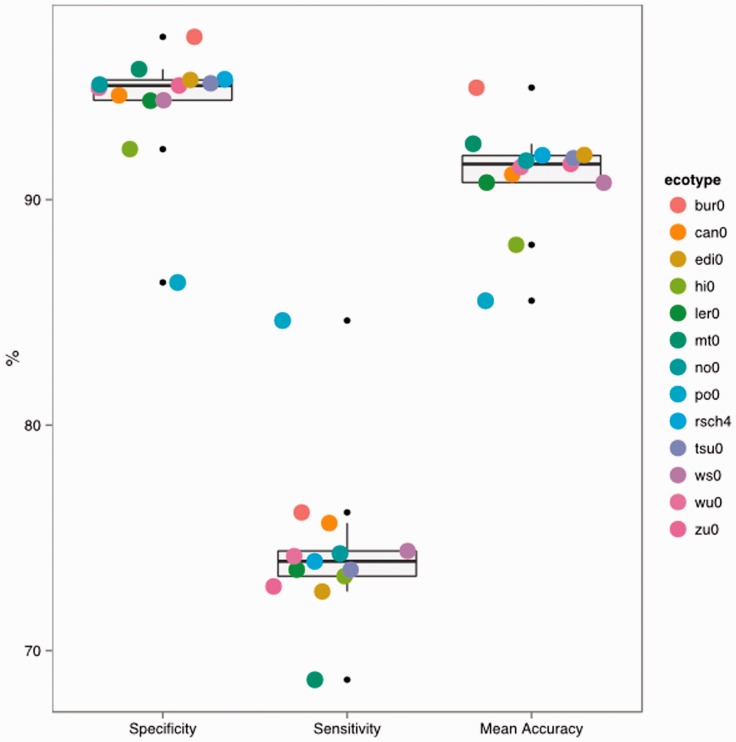
Classification accuracy of SNPs called using the Decision Tree algorithm to classify bubbles found in 2*k* + 2 searches on graphs made from sequence data of 13 ecotypes of *A.thaliana*. The results are the mean of 10 runs using the decision tree algorithm in WEKA (Hall 2009). Results are the mean of correctly classified bubbles that contain SNPs as listed in Gan *et al.* (2011)

We trained a decision tree to distinguish between known SNPs and non-SNP bubbles and applied it to the predicted bubbles from 16 ecotypes to successfully classify bubbles that represented real SNPs with higher accuracy whilst maintaining similar sensitivity and specificity than using bubble structure alone (Fig. 5). The specificity ranged between 97.22 and 86.33%, and sensitivities vary between 84.63 and 68.71% each of which is similar to the values seen when using structure alone. Accuracy in the predicted SNP set was from 86.33 to 97.04%, much higher than from structure. This result indicates that structure and extra bubble attributes can give very highly accurate sets of predicted SNP sets. In a similar approach, with very similar *A.thaliana* data Leggett *et al.* (2013) used a ranking heuristic to classify SNPs after initial prediction but found that accuracy of that heuristic was compromised as the number of SNPs predicted increased and in SNP sets of over 100 000 each newly predicted SNP has only a 40% likelihood of being accurate such that SNP sets of the size we predict contained very high numbers of false-positive SNP calls, the 2*k* + 2 algorithm and classifier outperforms that approach significantly returning better than 90% accurate SNP calls for very large SNP sets.

## 5 Conclusion

The 2*k* + 2 algorithm takes a graph theoretical approach to identifying topological structures, namely 2*k* + 2 cycles in undirected graphs that can represent SNPs in sequences input to a donor De Bruijn graph. We have shown that 2*k* + 2 can be used to generate sets of SNPs in graphs at high accuracy in the *A.thaliana* genome which is increased by application of the decision tree classifier. 2*k* + 2 found SNPs across all portions of the *A.thaliana* genome without bias towards feature type or region. We did not find that all SNPs seen in alignment-based methods using a reference could be detected with this algorithm and in all our experiments, we found that around 70% of SNPs seen by alignment could be predicted. The 2*k* + 2 strategy is, therefore, a useful algorithm that should find general use in reference-free SNP calling strategies in the future.

## Supplementary Material

Supplementary Data
