# Supplementary material for: Using 2k + 2 bubble searches to find single nucleotide polymorphisms in k-mer graphs
Source: Bioinformatics. 2014 Oct 24;31(5):642–6. doi: 10.1093/bioinformatics/btu706 (PMC4341063; doi:10.1093/bioinformatics/btu706)
Supplement: Supplementary Data [file supp_31_5_642__index.html]

Using 2k + 2 bubble searches to find Single Nucleotide Polymorphisms in k-mer graphs — Using 2k + 2 bubble searches to find single nucleotide polymorphisms in k-mer graphs — Using 2k + 2 bubble searches to find single nucleotide polymorphisms in k-mer graphs — Supplementary Data 

# Using 2*k* + 2 bubble searches to find single nucleotide polymorphisms in *k*-mer graphs

## Supplementary Data

files

**Files in this Data Supplement:**

- Supplementary Data - pdf file
