# Supplementary material for: Using 2k + 2 bubble searches to find single nucleotide polymorphisms in k-mer graphs
Source: Bioinformatics. 2014 Oct 24;31(5):642–6. doi: 10.1093/bioinformatics/btu706 (PMC4341063; doi:10.1093/bioinformatics/btu706)
Supplement: Supplementary Data [file supp_btu706_supplemental-data.pdf]

1 Supplemental Data

1.1 Training sets for decision trees

The abstraction of coverages for machine learning with Decision Trees is described as follow:

- Condition 1: coverages on one side of the bubble are above 0 and the other side are 0. We call this colored. assigned letter (o) and (g) respectively.
- Condition 2: coverages are above 0 on both paths of the bubble. We call this mixed. Assigned a letter (m)

The class positive (+) and negative (-) are produced using the blast algorithm and matched the SNPs genomic positions with those of list in [ref]. Positive bubbles are those that contained the SNP in the list and Negative bubbles are those that are not in the list.

Bubbles are separated into two sets by looking at coverages on each path. In Table S2, bubblescol is the number of bubbles that satisfy condition 1 and bubblesmix is the number of bubbles that satisfy condition 2 mentioned above.

We notice in Table S2 that the arabidopsis variants contain bubbles with different proportions. We added in the same table the number of blasted bubbles (Blastedbubbles) and the difference (diff) that shows the number of bubbles that could not be found in blast search. Although the size k set in this experiment is 31, only 1 bubble was not found using blast for 4 dataset.

The number of SNPs found four each bubble type (positivecol and positivemix) is shown in Table S3. SNPs found in colored bubbles are shown in positivemix while SNPs found in mixed bubbles are shown in positivemix. The accuracies are shown in matchcolacc and matchmixac for colored and mixed bubbles respectively.
The results in Table S3 show clearly that colored bubbles are more likely to contain real SNP than mixed bubbles. However, mixed bubbles also contain a large proportion of SNPs.

Our method shows that it is possible to use available SNP lists produced by other means to predict with high accuracy SNPs as found using graphs produced by Cortex Assembly package. As more SNPs are genotyped larger list are created thus helping find accurate patterns in bubbles as produced by corex. In the future, it is possible to separate the attributes and train classifiers to find the most discriminative attribute set. In addition, other classifiers can be used to find the best performing for these type of datasets.

Table S1: The datasets containing bubbles of various coverages. Total samples (bubbles) per dataset, positive (+) samples and negative (-) samples in the dataset, the trainset set is made of 2/3 of the dataset, and the testset is made of 1/3 of the dataset.

dataset	total	+ samples	- samples	trainset	testset
can0	118830	21946	96884	79220	39610
ler0	105536	18444	87092	70357	35179
edi0	117992	17529	100463	78661	39331
tsu0	109320	16834	92486	72880	36440
hi0	119261	26700	92561	79507	39754
wu0	103538	17469	86069	69025	34513
no0	116406	18833	97573	77604	38802
ws0	108445	19884	88601	72296	36149
zu0	100226	15771	84515	66817	33409
rsch4	113483	17931	95552	75655	37828
po0	225806	108111	117695	150537	75269
mt0	116152	14189	101963	77434	38718
bur0	151357	16186	135171	100904	50453

Table S2: $2K+2$ cycles (bubbles) of 13 variants of arabidopsis thaliana assembled genome using cortex. Blastedbubbles is the number of bubbles found in the reference genome Tair10 by blast algorithm using default parameters. totalbubbles is the total number of bubbles found by $2K+2$ algorithm. diff is the difference between the two i.e. Blastedbubbles vs totalbubbles. bubblescolored is the number of bubbles that have coverages > 0 on one path and 0 in the other path of the bubble. bubblesmixed is the number of bubbles that have mixed coverages in both paths

Datasets	Blastedbubbles	totalbubbles	diff	bubblescolored	bubblesmixed
can0	355501	355501	0	193341	162160
ler0	310545	310545	0	167418	143127
hi0	261360	261361	1	109641	151720
wu0	284160	284160	0	143857	140303
zu0	271424	271425	1	136773	134652
rsch4	303826	303827	1	148788	155039
tsu0	301916	301916	0	153336	148580
edi0	320868	320869	1	159982	160887
po0	421458	421458	0	130056	291402
no0	319898	319898	0	162826	157072
ws0	321396	321396	0	172944	148452
mt0	292070	292070	0	136626	155444
bur0	381589	381589	0	170250	211339

Table S3: $2K + 2$ cycles (bubbles) of 13 variants of arabidopsis thaliana assembled genome using cortex. positivecol is the number of bubbles that have coverages > 0 on one path and 0 in the other path which matched a SNP in the list of SNPs [Xiangchao *et al.*, 2011]. positivemix is the number of bubbles that have mixed coverages wich matched SNPs in the lists of SNPs. matchcolacc is the accuracy in % of SNPs found in bubbles from the bubblescolored and matchmixacc is the accuracy in % of SNPs found in bubbles from the bubblesmixed

dataset	positivecol	positivemix	matchcolacc %	matchmixacc %
can0	168621	21946	87.21	13.53
ler0	145950	18444	87.18	12.89
hi0	88247	26700	80.49	17.60
wu0	125588	17469	87.30	12.45
zu0	116027	15711	84.83	11.67
rsch4	129143	17931	86.80	11.57
tsu0	134483	16834	87.70	11.33
edi0	139792	17529	87.38	10.90
po0	101388	108111	77.96	37.10
no0	141874	18833	87.13	11.99
ws0	148088	19844	85.63	13.37
mt0	109493	14189	80.14	9.13
bur0	138094	16186	81.11	7.66

Table S4: Classification accuracies of 13 variants of arabidopsis thaliana assembled genome using cortex. The results are the mean of 10 runs using the decision tree algorithm in WEKA [Mark *et al.*, 2009]. Avgacc is the mean of correctly classified bubbles that contains SNPs as listed in their respective SNP lists [Xiangchao *et al.*, 2011]. AvgTP is the mean true positive, avgFP is the mean false positive, avgTN is the mean true negative, AvgFN is the mean false negative, specificity, sensitivity and the mean accuracy are in percentage

dataset	avgacc	avgTP	avgFP	avgTN	avgFN	specificity %	sensitivity %	avgacc %
can0	36093.50	5534.20	1735.70	30559.30	1780.80	94.63	75.66	91.12
ler0	31927.00	4524.40	1628.40	27402.60	1623.60	94.39	73.59	90.76
hi0	34983.10	6523.80	2394.70	28459.30	2376.20	92.24	73.30	88.00
wu0	31563.70	4319.90	1446.20	27243.80	1503.10	94.96	74.19	91.45
zu0	30595.70	3815.20	1391.50	26780.50	1421.80	95.06	72.85	91.58
rsch4	34785.70	4420.50	1485.80	30365.20	1556.50	95.34	73.96	91.96
tsu0	33465.50	4128.80	1492.10	29336.70	1482.40	95.16	73.58	91.84
edi0	36175.50	4243.40	1569.70	31918.30	1599.60	95.31	72.62	91.98
po0	64370.50	30500.00	5361.50	33870.50	5537.00	86.33	84.64	85.52
no0	35594.60	4663.90	1594.10	30930.70	1613.30	95.10	74.30	91.73
ws0	32806.90	4922.80	1649.90	27884.10	1692.20	94.41	74.42	90.75
mt0	35807.40	3249.90	1430.50	32557.50	1480.10	95.79	68.71	92.48
bur0	47914.50	4108.00	1250.50	43806.50	1288.00	97.22	76.13	94.97

branching nodes in subgraph	ecotype	snps	proportion
2	can0	26824	0.491138128
2	bur0	12901	0.368147704
2	po0	15774	0.432365759
3	can0	17094	0.312985206
3	bur0	11509	0.328425078
3	po0	11882	0.325685936
4	can0	6655	0.12185074
4	bur0	5795	0.165368262
4	po0	5371	0.147219253
5	can0	2554	0.046762853
5	bur0	2633	0.075136261
5	po0	2039	0.055889044
6	can0	1236	0.022630731
6	bur0	1222	0.034871444
6	po0	920	0.025217224
7	can0	711	0.013018163
7	bur0	686	0.01957595
7	po0	529	0.014499904
8	can0	411	0.007525267
8	bur0	406	0.011585766
8	po0	327	0.008963079
9	can0	284	0.005199941
9	bur0	229	0.006534829
9	po0	212	0.005810926
10	can0	191	0.003497144
10	bur0	172	0.004908256
10	po0	140	0.003837404
11	can0	160	0.002929544
11	bur0	120	0.003424364
11	po0	102	0.002795823
12	can0	91	0.001666178
12	bur0	81	0.002311446
12	po0	72	0.001973522
13	can0	83	0.001519701
13	bur0	65	0.001854864
13	po0	36	0.000986761
14	can0	46	0.000842244
14	bur0	31	0.000884627
14	po0	32	0.000877121
15	can0	26	0.000476051
15	bur0	20	0.000570727
15	po0	15	0.00041115
16	can0	28	0.00051267
16	bur0	13	0.000370973
16	po0	12	0.00032892
17	can0	12	0.000219716
17	bur0	11	0.0003139
17	po0	11	0.00030151

18	can0	12	0.000219716
18	bur0	9	0.000256827
18	po0	5	0.00013705
19	can0	5	9.15483E-05
19	bur0	3	8.56091E-05
19	po0	3	8.22301E-05
20	can0	7	0.000128168
20	bur0	4	0.000114145
20	po0	5	0.00013705
21	can0	1	1.83097E-05
21	bur0	5	0.000142682
21	po0	2	5.48201E-05
22	bur0	1	2.85364E-05
22	po0	1	2.741E-05
23	can0	1	1.83097E-05
23	bur0	1	2.85364E-05
24	can0	1	1.83097E-05
25	po0	1	2.741E-05
26	can0	1	1.83097E-05
29	can0	1	1.83097E-05

Table S5: Summary of branching nodes and proportion of total subgraphs made up with a given number of branching nodes in subgraphs with 2 or more branched nodes

Table S6: Number of SNPs called and found in four canonical SNP lists by the Cortex assembler [Iqbal *et al.*, 2012]

Ecotype	Variants predicted by Cortex	Variants found in SNP List	Variants not found in SNP list
can0	165276	153706	11570
edi0	144558	138246	6312
hi0	128125	119412	8713
ler0	18563	17077	1486

References

[Altschul *et al.*, 2000] Altschul, S.F., Gish, W., Miller, W., Myers, E.W., Lipman, D.J. (1990). Basic local alignment search tool, *Journal of Molecular Biology*, **215**, 403-410.

[Iqbal *et al.*, 2012] Iqbal, Z., Caccamo, M., Turner, I., Flicek, P., McVean, G. (2012). De novo assembly and genotyping of variants using colored de Bruijn graphs , *Nature Genetics*, **44**, 226-232.

[Li and Durbin, 2009] Li, H., Durbin, R. (2009). Fast and accurate short read alignment with Burrows-Wheeler transform. *Bioinformatics*, **25**, 1754-1760.

[Leggett and MacLean, 2014] Leggett, R.M., MacLean, D. (2014) Reference-free SNP detection: Dealing with the data deluge *BMC Genomics*, *In Press.*

[Li *et al.*, 2009] Li, H., Handsaker, B., Wysoker, A., Fennell, T., Ruan, J., Homer, N., Marth, G., Abecasis, G., Durbin, R. (2009). The Sequence Alignment/Map format and SAMtools. *Bioinformatics*, **25**, 2078-2079.

[Schneeberger *et al.*, 2011] Schneeberger, K., Ossowski, S., Ott, F., Klein, J.D., Wang, X., Lanz, C., Smith, L.M., Cao, J., Fitz, J., Warthmann, N., Henz, S.R., Huson, D.H., Weigel, D. (2011). Reference-guided assembly of four diverse Arabidopsis thaliana genomes. *Proc Natl Acad Sci USA*, **108**, 102459-10254.

[Zerbino and Birney, 2008] Zerbino, D. R., Birney, E. (2008). Velvet: algorithms for de novo short read assembly using de Bruijn graphs, *Genome Research*, **18**, 821-829.

[Floyd, 1967] Robert W Floyd.(1967). Nondeterministic Algorithms. *J. ACM*, **14, 4**, 636-644.

[Leggett *et al.*, 2013] Leggett, R.M., Ramirez-Gonzalez, R.H., Verweij, W., Iqbal, Z., Jones, J.D.G., Caccamo, M., MacLean, D. (2003) Identifying and classifying trait linked polymorphisms in non-reference species by walking coloured De Bruijn graphs, *submitted* .

[Pevzner *et al.*, 2001] Pevzner, P. A., Tang, H., Waterman, M. S. (2001). An Eulerian path approach to DNA fragment assembly. *Proc Natl Acad Sci USA*, **98**, 9748-9753.

[Simpson *et al.*, 2009] Simpson, J. T., Wong, K., Jackman, S. D., Schein, J. E., Jones, S. J. M., Birol, I. (2009). ABySS: a parallel assembler for short read sequence data, 19(6), 1117-23. *Genome Research*, **19**, 1117-1123.

[Peterlongo *et al.*, 2010] Peterlongo, P., Schnel N., Pisanti N., Sagot, M.F., Lacroix, V. (2010) Identifying SNPs without a Reference Genome by Comparing Raw Reads *Lecture Notes in Computer Science*, **6393**, 147-158.

[AGI, 2000] The Arabidopsis Genome Initiative (2000) Analysis of the genome sequence of the flowering plant Arabidopsis thaliana, *Nature*, **408**, 796-815.

[Quinlan, 1986] Induction of decision trees (1986) Quinlan, J. Ross. Machine learning 1.1:81-106.

[Mark *et al.*, 2009] Hall, Mark, et al. "The WEKA data mining software: an update." ACM SIGKDD explorations newsletter 11.1 (2009): 10-18.

[Xiangchao *et al.*, 2011] Gan, Xiangchao, et al. "Multiple reference genomes and transcriptomes for Arabidopsis thaliana." Nature 477.7365 (2011): 419-423.
